# Adaptation of antibiotics and antifungal strategy to preoperative biliary drainage to improve postoperative outcomes after pancreatic head resection

**DOI:** 10.1002/wjs.12446

**Published:** 2024-12-16

**Authors:** Fabio Giannone, Charles Lagarrigue, Oronzo Ligurgo, Lina Jazaerli, Paul Michel Mertes, Olivier Collange, Patrick Pessaux

**Affiliations:** ^1^ Department of Visceral and Digestive Surgery University Hospital of Strasbourg Strasbourg France; ^2^ Inserm Institut de Recherche sur Les Maladies Virales et Hépatiques U1110 Strasbourg University Strasbourg France; ^3^ Hepato‐Pancreato‐Biliary, Oncologic and Robotic Unit Azienda Ospedaliero‐Universitaria SS. Antonio e Biagio e Cesare Arrigo Alessandria Italy; ^4^ Department of Anesthesiology and Intensive Care University Hospital of Strasbourg Strasbourg France

**Keywords:** antibiotics, biliary drainage, outcomes, pancreatic cancer

## Abstract

**Background:**

Biliary contamination significantly correlates with major comorbidities during pancreatic head resection. Recently, a piperacillin‐tazobactam prophylaxis demonstrated a lower rate of infectious complications (IC) and postoperative pancreatic fistula (POPF) in this population. However, bacterial contamination is rare in patients without a preoperative biliary drainage (PBD) and probably could not benefit from this antibiotic. Furthermore, little is known about the role of biliary fungal contamination.

**Method:**

All retrospective cases undergoing pancreatic head resection with intraoperative biliary sample were included. Postoperative outcomes of patients with a piperacillin‐tazobactam‐based treatment were compared to cases in which a narrow‐spectrum antibiotic was administrated, stratified according to the use of a PBD. The same analysis was repeated for antifungal treatment administration.

**Results:**

Among the 205 cases included, PBD was necessary in 127 patients (62%). Broad‐spectrum treatment was associated with fewer overall and clinically relevant POPF (*p* = 0.001 and *p* = 0.004), overall morbidity (*p* = 0.044), and overall IC (*p* = 0.018), but only in the PBD group. Similarly, antifungal treatment was significantly associated with some specific IC only in the PBD group. At multivariable analysis, antifungal therapy in the whole cohort (*p* = 0.029) and the use of a piperacillin‐tazobactam (*p* = 0.007) treatment in patients with a PBD were independently associated with a reduced risk of a clinically relevant POPF.

**Conclusions:**

A broad‐spectrum antibiotic therapy reduces overall morbidity after pancreatic head resection, but only in cases with a history of PBD. Furthermore, the use of an antifungal prophylaxis or therapy should be further investigated in these patients because it may reduce the risk of some IC.

## Graphical abstract

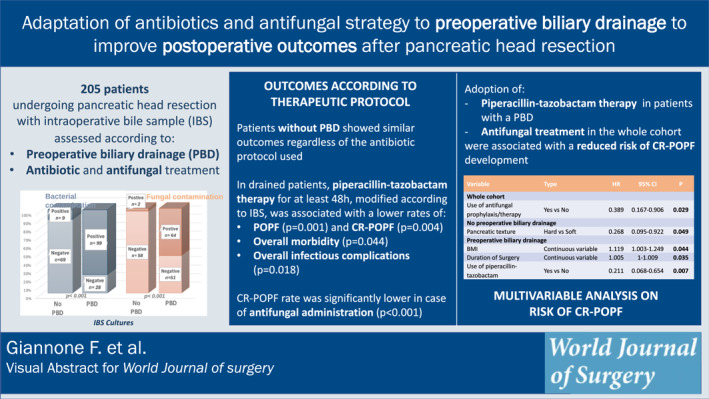

## 1 INTRODUCTION

The high morbidity observed after pancreatoduodenectomy (PD) has always been a long‐standing problem in pancreatic surgery with a reported postoperative complications rate of up to 60% in large series.^1^, ^2^ Postoperative pancreatic fistula (POPF) and infectious comorbidities are certainly the most challenging issues in the perioperative care, being both of them major determinants of outcomes.^3^, ^4^ Bacteriobilia, in particular, in addition to the widely described association with surgical site infection (SSI),^5^, ^6^ has been largely correlated with the development and with the grade of POPF.^7^, ^8^, ^9^, ^10^, ^11^, ^12^ Different studies reported the correlation between the germs found in the intraoperative bile samples (IBS) and postoperative cultures and, recently, results from a randomized controlled trial (RCT) suggested that a significant reduction in SSI and clinically relevant POPF (CR‐POPF) can be achieved by switching cephalosporin for piperacillin‐tazobactam‐based antibiotic prophylaxis before PD.^13^ This is probably a consequence of the resistance profile of the bacteria usually found in biliary cultures, which are often resistant to cephalosporines.^14^, ^15^ Preoperative biliary drainage (PBD) is undoubtedly the most important determinant of biliary contamination in this scenario.^16^, ^17^, ^18^, ^19^, ^20^, ^21^, ^22^, ^23^ Although a correct prophylaxis seems to be established, the use of postoperative therapy is controversial, being suggested by the enhanced recovery after surgery (ERAS) guidelines and by some series,^24^, ^25^, ^26^, ^27^ but not always supported by literature in terms of postoperative outcomes. Furthermore, no clear indication on the type of antibiotic to adopt as a systematic postoperative therapy exists. Similarly, another debate concerns perioperative antifungal treatment. This issue is limited by the relatively poor data concerning the incidence and severity of fungal biliary contamination. In any case, here again, this is strongly correlated with preoperative biliary procedures and drainage.^28^, ^29^, ^30^ Some series reported a correlation between postoperative complications and fungal contamination.^28^, ^31^ It is likely that, as it happened for bacterial contamination, the presence of fungi in IBS could influence the morbidity rate after PD and, at the same time, a perioperative treatment should be implemented in selected patients.^32^

The aim of this manuscript was twofold. First, we assessed the importance of the type of antibiotic treatment in terms of POPF and other postoperative complications, focusing at the same time on the possibility to select patients who will benefit from this therapy according to the presence of a biliary stent. Furthermore, the impact of fungal contamination and antimycotic therapy on short‐term outcomes was assessed through the same analysis.

## 2 MATERIAL AND METHODS

This is a retrospective mono‐institutional study conducted according to the strengthening the reporting of observational studies in epidemiology guidelines.^33^ An informed consent was obtained before each procedure and the study was aligned to the ethical standards of the Helsinki declaration.^34^ All consecutive adult patients (age ≥18 years) undergoing PD or total pancreatectomy (TP) from 2014 to 2022 were initially included in the analysis. Since January 2014, an IBS of both gallbladder and common bile duct was collected in routine practice during all PD and TP. This sample was analyzed for bacterial and fungal contamination, regardless of preoperative history, comorbidities, or drainage procedures. Metastatic patients, surgery not performed and cases in which a bile sample was not collected for any reason were excluded from the initial cohort. Patients who died during or at most 48 h after surgery from noninfectious causes were as well excluded.

### 2.1 Perioperative antibiotic care and surgical procedure

In patients undergoing surgery before 2019, antibiotic and antimycotic prophylaxis and therapy were chosen case by case by the surgeon and the anesthetist according to patients “previous history without any type of standardization.” Since 2019, an internal protocol has been established and strictly implemented in clinical practice in our institution following the recommendation of the French Society of Anesthesiology and Intensive Care.^35^ The protocol was mainly centered on the presence or not of a PBD and the rational lies on the frequent absence of biliary contamination in patients without any biliary gestures, whereas drained patients often presented resistant bacteria and fungi which were responsible for a higher postoperative morbidity.^18^, ^28^, ^29^, ^30^ In detail, patients without any type of PBD received an exclusive prophylaxis with an intravenous administration of 2 g dose of cefoxitin (or cefazolin) and 500 mg of metronidazole, 30 min before skin incision. The same dose was repeated every 2 h for the cephalosporin. A cephalosporin was preferred to penicillin/beta‐lactamase inhibitors because of the occasional presence of resistant *Escherichia coli* in the bile samples of these patients.^35^ No antifungal prophylaxis was administrated and patients did not receive any postoperative antibiotic or antifungal therapy. On the contrary, patients who were preoperatively drained because of a hyperbilirubinemia, regardless of the method used, received an intravenous prophylaxis of piperacillin‐tazobactam (3.375 or 4.5 g) and caspofungin (70 mg), 30 min before skin incision. The same dose was repeated every 6 h for piperacillin‐tazobactam. A single shot of gentamicin (or amikacin) was also administrated. A postoperative administration of 16 g/day of piperacillin/tazobactam and 50 mg/day of caspofungin was maintained in these patients. These drugs were adapted to body weight, allergies, renal failure, or other conditions which needed any modification of the protocol. Biliary cultures were checked after 48–72 h and, according to the pathogens found and their antibiogram, an adequate therapy was introduced/modified if necessary for a total of 7 days, or, in case of negative culture, therapy was discontinued. As regard to a surgical procedure, a cephalic or total duodenopancreatectomy without pyloric conservation was performed, associated with a locoregional lymphadenectomy. A pancreatico‐gastric and a hepatico‐jejunal anastomosis were performed. An external trans‐anastomotic stent was placed in case of high‐risk pancreatic anastomosis, exteriorized in the anterior face of the stomach and then through the abdominal wall. The abdominal cavity was drained with two blades, one close to the biliary anastomosis and another protecting the pancreatic anastomosis.

### 2.2 Outcomes and statistical analysis

The cohort was divided into two groups according to the perioperative antibiotic care: (i) a first group considered all cases receiving an antibiotic prophylaxis of piperacillin‐tazobactam and at least 48/72 h of the same drug, as in our internal protocol; (ii) the second group was instead made up of patients receiving a prophylaxis or a perioperative therapy with a narrower‐spectrum antibiotic, as a first or second generation of cephalosporin with or without a nitroimidazole (metronidazole). These two cohorts were compared to assess the development of overall and specific 90‐days complications, which was our primary aim. This analysis was stratified according to the presence of a PBD to evaluate the superiority of a piperacillin‐tazobactam‐based antibiotic care in the two groups. The same analysis was then repeated to assess the utility of a fungal treatment, dividing the whole cohort into patients receiving or not an antifungal prophylaxis or therapy. A multivariable analysis to assess the main predictors of CR‐POPF was then performed. We finally analyzed bacterial and fungal species found in IBS to determine the most frequent germs, as well as their resistance to classic antibiotic therapy. The pancreatic texture was evaluated by the first surgeon and prospectively classified as soft/hard in our institutional database. Postoperative complications were stratified according to the Clavien–Dindo classification.^36^, ^37^ SSI was defined as the presence of purulent or infected liquid drained from the skin (superficial SSI) or inside the abdominal cavity (deep SSI) with or without the need for drainage.^38^ Pancreatic‐specific complications were classified according to the International Study Group in Pancreatic Surgery guidelines.^39^, ^40^, ^41^

All categorical data are displayed with relative proportions (%), with distribution between groups assessed using the chi^2^ Pearson test. Yates's correction and Fisher exact test were used when necessary. Continuous data are reported as median with interquartile range, and comparisons between subgroups were carried out using the student's *t*‐test or Mann–Whitney *U* test in case of normal distribution. Univariate and multivariable analyses were performed using binary logistic regression. Variables with *p* < 0.1 in the univariate analysis were included in the final multivariable analysis model. All tests were two‐tailed, and the level of significance was set at *p* < 0.05. All statistical computations were performed using SPSS (version 26.0; SPSS Statistics for Macintosh, IBM Corp).

## 3 RESULTS

### 3.1 Study cohort

After excluding those patients who did not meet the inclusion criteria, a total of 205 patients were available in the final cohort. A complete flowchart of the selection process is shown in Supporting Information S1: Online Resource 1. PBD was necessary in 127 patients (62%) of whom 105 endoscopic (82.7%) and 22 percutaneous (17.3%), including 7 cases (5.5%) in which a percutaneous biliary drainage was placed after endoscopic failure. All patients had a serum level of bilirubin inferior to 150 μmol/L at surgical resection. Table 1 shows main pre and perioperative variables according to the antibiotic and antifungal protocol used. As regard to the use of piperacillin‐tazobactam, the population was similar in almost all clinical and surgical variables, except for a higher use of this type of antibiotic in the PBD group (*p* < 0.001), in patients with a pancreatic ductal adenocarcinoma (PDAC) or a duodenal/ampullary cancer (*p* = 0.001) as well as in patients undergoing neoadjuvant therapy (*p* = 0.024). Antifungal treatment was mostly used in patients with PBD (*p* < 0.001) in case of PDAC (*p* = 0.001) and when preoperative antibiotics were used (*p* = 0.046). Its use was also higher in patients with a harder pancreatic texture (*p* = 0.040).

**TABLE 1:** General features of the overall cohort according to the use of a piperacillin‐tazobactam perioperative therapy (*Antibiotic treatment*) and an antifungal prohylaxis/therapy (*Antifungal treatment*).

Variable	Antibiotic treatment		*p*	Antifungal treatment		*p*
	No piperacillin‐tazobactam, *n* = 88	Piperacillin‐tazobactam, *n* = 117		No prophylaxis/therapy, *n* = 70	Antifungal prophylaxis/therapy, *n* = 135	
	*n* (%)			*n* (%)		
Sex						
Male	54 (61.4)	73 (62.4)	0.881	43 (61.4)	84 (62.2)	0.912
Female	34 (38.6)	44 (37.6)		27 (38.6)	51 (37.8)	
Age, median (IQR)	66 (58–72)	66 (60–75)	0.968	66 (59–72)	67 (60–75)	0.558
BMI						
<25	44 (50)	65 (55.6)	0.430	35 (50)	74 (54.8)	0.512
≥25	44 (50)	52 (44.4)		35 (50)	61 (45.2)	
Weight loss >10%						
No	60 (68.2)	91 (77.8)	0.123	52 (74.3)	99 (73.3)	0.883
Yes	28 (31.8)	26 (22.2)		18 (25.7)	36 (26.7)	
ASA						
I	10 (11.4)	4 (3.4)	0.069	7 (10)	7 (5.2)	0.245
II	40 (45.5)	63 (53.8)		31 (44.3)	72 (53.3)	
III	38 (43.2)	50 (42.7)		32 (45.7)	56 (41.5)	
Diabetes						
No	63 (71.6)	75 (64.1)	0.258	47 (67.1)	91 (67.4)	0.969
Yes	25 (28.4)	42 (35.9)		23 (32.9)	44 (32.6)	
Respiratory diseases						
No	77 (87.5)	97 (82.9)	0.363	62 (88.6)	112 (83)	0.391
Yes	20 (17.1)	20 (17.1)		8 (11.4)	23 (17)	
Cardiovascular diseases						
No	35 (39.8)	58 (49.6)	0.163	33 (47.1)	60 (44.4)	0.713
Yes	53 (60.2)	59 (50.4)		37 (52.9)	75 (55.6)	
Chronic renal failure						
No	80 (90.9)	103 (88)	0.510	60 (85.7)	123 (91.1)	0.236
Yes	8 (9.1)	14 (12)		10 (14.3)	12 (8.9)	
Preoperative ICU hospitalization						
No	83 (94.3)	110 (94.1)	0.928	66 (94.3)	127 (94.1)	0.951
Yes	5 (5.7)	12 (5.9)		4 (5.7)	8 (5.9)	
Preoperative antibiotics						
No	72 (81.8)	94 (80.3)	0.790	62 (88.6)	104 (77)	*0.046*
Yes	16 (18.2)	23 (19.7)		8 (11.4)	31 (23)	
Neoadjuvant therapy						
No	79 (89.8)	91 (77.8)	*0.024*	63 (90)	107 (79.3)	0.053
Yes	9 (10.2)	26 (22.2)		7 (10)	28 (20.7)	
Preoperative biliary drainage						
No	56 (63.6)	22 (18.8)	*<0.001*	49 (70)	29 (21.5)	*<0.001*
Yes	32 (36.4)	95 (81.2)		21 (30)	106 (78.5)	
Type of resection						
PD	4 (4.5)	5 (4.3)	0.925	3 (4.3)	6 (4.4)	0.958
TP	84 (95.5)	112 (95.7)		67 (95.7)	129 (95.6)	
Type of tumor						
PDAC	45 (51.5)	75 (64.1)	*0.001*	35 (50)	85 (63)	*0.001*
Ampullary/duodenal	18 (20.5)	26 (22.2)		14 (20)	30 (22.2)	
DCCA	2 (2.3)	8 (6.8)		1 (1.4)	9 (6.7)	
Other	23 (26.1)	8 (6.8)		20 (28.6)	11 (8.1)	
Pancreatic texture^a^						
Soft	51 (60.7)	53 (49.1)	0.108	43 (65.2)	61 (48.4)	*0.040*
Hard	33 (39.3)	55 (50.9)		23 (34.8)	65 (51.6)	
DOS (min), median (IQR)	560 (480–630)	540 (460–600)	0.481	557 (420–647)	540 (480–600)	0.578
EBL (mL), median (IQR)	500 (300–800)	400 (250–600)	0.263	445 (265–762)	400 (265–700)	0.898

*Note*: Italic values indicate the significative correlation.

Abbreviations: ASA, American society of anesthesiologists; BMI, body mass index; dCCA, distal cholangiocarcinoma; DOS, duration of surgery; EBL, estimated blood loss; ICU, intensive care unit; IQR, interquartile range; PD, pancreatoduodenectomy; PDAC, pancreatic ductal adenocarcinoma; TP, total pancreatectomy.

^a^ Four cases missing and 9 cases not considered due to the absence of a pancreatic anastomosis (total pancreatectomy).

### 3.2 Postoperative outcomes according to therapeutic protocol

Postoperative outcomes were compared according to the use of piperacillin‐tazobactam, separately for patients receiving or not a PBD (Table 2). Patients without PBD showed similar outcomes regardless of the antibiotic protocol used. On the contrary, drained patients treated with a large‐spectrum antibiotic had a significantly lower risk of some pancreatic‐specific and infectious complications (ICs) as fewer POPF (*p* = 0.001), CR‐POPF (*p* = 0.004), overall morbidity (*p* = 0.044), and overall ICs (*p* = 0.018). The second analysis compared patients receiving or not an antifungal prophylaxis or therapy (Table 3). CR‐POPF rate was significantly lower in case of antifungal administration (*p* < 0.001) in the whole cohort, but it did not reach the significance in the PBD group. Patients receiving this treatment showed also fewer rates of some ICs in case of a PBD as urinary infections (*p* < 0.001), pneumoniae (*p* = 0.012), and less consequent acute respiratory failure (*p* = 0.040).

**TABLE 2:** Postoperative complications comparison between patients undergoing antibiotic prophylaxis and therapy according with or without pieperacillin‐tazobactam, stratified for preoperative biliary drainage in the whole cohort.

Variables	No preoperative biliary drainage		*p*	Preoperative biliary drainage		*p*
	No piperacillin‐tazobactam *n* = 56	Piperacillin‐tazobactam *n* = 22		No piperacillin‐tazobactam *n* = 32	Piperacillin‐tazobactam, *n* = 95	
	*n* (%)			*n* (%)		
Postoperative morbidity	48 (85.7)	15 (68.2)	0.147	27 (84.4)	60 (63.2)	*0.044*
Major complications (CD > 2)	18 (32.1)	6 (27.3)	0.883	9 (28.1)	20 (21.1)	0.561
Postoperative death	3 (5.4)	0	0.555	2 (6.3)	5 (5.3)	1
Pancreatic‐specific complications						
POPF^a^	23 (43.4)	7 (35)	0.701	15 (48.4)	15 (16.3)	*0.001*
CR‐POPF^a^	19 (35.8)	5 (25)	0.548	11 (35.5)	10 (10.9)	*0.004*
Postoperative hemorrhage	14 (25)	4 (18.2)	0.730	7 (21.9)	12 (12.6)	0.326
Delayed gastric emptying	23 (41.1)	9 (40.9)	1	12 (37.5)	24 (25.3)	0.271
Chyle leak	2 (3.6)	0	1	1 (3.1)	5 (5.3)	0.991
Infectious complications						
Overall infectious complications	34 (60.7)	9 (40.9)	0.184	19 (59.4)	32 (33.7)	*0.018*
Overall SSI	19 (33.9)	4 (18.2)	0.273	8 (25)	14 (14.7)	0.291
Superficial SSI	12 (21.4)	2 (9.1)	0.342	6 (18.8)	11 (11.6)	0.465
Deep SSI	11 (19.6)	4 (18.2)	1	4 (12.5)	9 (9.5)	0.880
Bacteriemia	15 (26.8)	3 (13.6)	0.346	8 (25)	12 (12.6)	0.167
Cholangitis	3 (5.4)	1 (4.5)	1	3 (9.4)	5 (5.3)	0.415
CVC Infection	6 (10.7)	1 (4.5)	0.676	7 (21.9)	6 (6.3)	*0.030*
Urinary infection	6 (10.7)	3 (13.6)	1	4 (12.5)	2 (2.1)	*0.035*
Pneumonia	5 (8.9)	1 (4.5)	0.856	6 (18.8)	4 (4.2)	*0.024*
*Clostidrium* colitis	0	0	‐	2 (6.3)	0	0.062
Postoperative septic shock	7 (12.5)	1 (4.5)	0.530	5 (15.6)	13 (13.7)	1
Noninfectious complications						
Acute coronary syndrome	1 (1.8)	0	1	0	0	‐
Atrial fibrillation	3 (5.4)	0	0.555	2 (6.3)	7 (7.4)	1
Acute renal failure	9 (16.1)	1 (4.5)	0.320	5 (15.6)	13 (13.7)	1
Acute respiratory failure	5 (8.9)	1 (4.5)	0.856	4 (12.5)	8 (8.4)	0.739
Pulmonary embolism	1 (1.8)	0	1	1 (3.1)	1 (1.1)	0.442
Operative management						
Percutaneous drainage	10 (17.9)	1 (4.5)	0.247	3 (90.6)	9 (9.5)	1
Radiologic treatment	15 (26.8)	3 (13.6)	0.346	3 (9.4)	13 (13.7)	0.743
Reintervention	6 (10.7)	3 (13.6)	1	4 (12.5)	7 (7.4)	0.597

*Note*: Italic values indicate the significative correlation.

Abbreviations: CD, Clavien–Dindo; CR‐POPF, clinically‐relevant postoperative pancreatic fistula; CVC, central venous catheter; POPF, postoperative pancreatic fistula; SSI, surgical site infection.

^a^ Calculated after excluding 9 total pancreatectomies.

**TABLE 3:** Comparison of postoperative outcomes between patients undergoing or not antifungal prophylaxis or therapy stratified for preoperative biliary drainage in the whole cohort.

Variables	No preoperative biliary drainage		*p*	Preoperative biliary drainage		*p*
	No antifungal prophylaxis/therapy, *n* = 49	Antifungal prophylaxis/therapy, *n* = 29		No antifungal prophylaxis/therapy, *n* = 21	Antifungal prophylaxis/therapy, *n* = 106	
	*n* (%)			*n* (%)		
Postoperative morbidity	39 (79.6)	24 (82.8)	0.964	15 (71.4)	72 (67.9)	0.953
Major complications (CD > 2)	16 (32.7)	8 (27.6)	0.830	6 (28.6)	23 (21.7)	0.688
Postoperative death	2 (4.1)	1 (3.4)	1	3 (14.3)	4 (3.8)	0.160
Pancreatic‐specific complications						
POPF^a^	22 (47.8)	8 (29.6)	0.201	8 (38.1)	22 (21.6)	0.185
CR‐POPF^a^	19 (41.3)	5 (18.5)	0.081	7 (33.3)	14 (13.7)	0.063
Postoperative hemorrhage	11 (22.4)	7 (24.1)	1	5 (23.8)	14 (13.2)	0.363
Delayed gastric emptying	19 (38.8)	13 (44.8)	0.774	8 (38.1)	28 (26.4)	0.412
Chyle leak	1 (2)	1 (3.4)	1	1 (4.8)	5 (4.7)	1
Infectious complications						
Overall infectious complications	29 (59.2)	14 (48.3)	0.484	11 (52.4)	40 (37.7)	0.314
Overall SSI	17 (34.7)	6 (20.7)	0.292	4 (19)	18 (17)	1
Superficial SSI	10 (20.4)	4 (13.8)	0.667	3 (14.3)	14 (13.2)	1
Deep SSI	11 (22.4)	4 (13.8)	0.522	2 (9.5)	11 (10.4)	1
Bacteriemia	15 (30.6)	3 (10.3)	0.076	5 (23.8)	15 (14.2)	0.323
Cholangitis	2 (4.1)	2 (6.9)	0.625	1 (4.8)	7 (6.6)	1
CVC infection	5 (10.2)	2 (6.9)	0.933	5 (23.8)	8 (7.5)	0.064
Urinary infection	7 (14.3)	2 (6.9)	0.535	5 (23.8)	1 (0.9)	*<0.001*
Pneumonia	4 (8.2)	2 (6.9)	1	5 (23.8)	5 (4.7)	*0.012*
*Clostidrium* colitis	0	0	‐	2 (9.5)	0	*0.026*
Postoperative septic shock	4 (8.2)	4 (13.8)	0.461	4 (19)	14 (13.2)	0.720
Noninfectious complications						
Acute coronary syndrome	1 (2)	0	1	0	0	‐
Atrial fibrillation	2 (4.1)	1 (3.4)	1	0	9 (8.5)	0.358
Acute renal failure	7 (14.3)	3 (10.3)	0.879	4 (19)	14 (13.2)	0.720
Acute respiratory failure	4 (8.2)	2 (6.9)	1	5 (23.8)	7 (6.6)	*0.040*
Pulmonary embolism	0	1 (3.4)	0.789	1 (4.8)	1 (0.9)	0.304
Operative management						
Percutaneous drainage	9 (18.4)	2 (6.9)	0.285	4 (19)	8 (7.5)	0.216
Radiologic treatment	13 (26.5)	5 (17.2)	0.507	3 (14.3)	13 (12.3)	1
Reintervention	4 (8.2)	5 (17.2)	0.397	4 (19)	7 (6.6)	0.153

*Note*: Italic values indicate the significative correlation.

Abbreviations: CD, Clavien–Dindo; CR‐POPF, clinically‐relevant postoperative pancreatic fistula; CVC, central venous catheter; POPF, postoperative pancreatic fistula; SSI, surgical site infection.

^a^ Calculated after excluding 9 total pancreatectomies.

### 3.3 Multivariable analysis for CR‐POPF

Independent predictors for CR‐POPF were evaluated by starting from all the pre and perioperative variables included in the database. The analysis was performed for the whole cohort and subsequently according to the use of a PBD (Table 4). When assessing the general population, the only independent predictor of CR‐POPF was the administration of an antifungal prophylaxis or therapy (HR: 0.389, 95% CI: 0.167–0.906; *p* = 0.029). In patients without any PBD, the only variable found after multivariable analysis was the pancreatic texture (HR: 0.268, 95% CI: 0.095–0.922; *p* = 0.049). Finally, in drained patients, the risk of a CR‐POPF was independently associated with BMI (HR: 1.119, 95% CI: 1.1003–1.249; *p* = 0.044), duration of surgery (HR: 1.005, 95% CI: 1–1.009; *p* = 0.035), and use of piperacillin‐tazobactam (HR: 0.211, 95% CI: 0.068–0.654; *p* = 0.007) but not with the antifungal treatment.

**TABLE 4:** Multivariable analysis on the risk of development of a clinically relevant postoperative pancreatic fistula in the whole cohort of 196 patients (excluding 9 total pancreatectomies) and according to preoperative biliary drainage.

Variable	Type	HR	95% CI	*p*
Whole cohort				
Age	Continuous variable	0.984	0.955–1.014	0.294
BMI	Continuous variable	1.073	0.993–1.158	0.075
Preoperative biliary drainage	Yes versus no	1.064	0.422–2.682	0.895
Type of tumor	PDAC versus other	0.542	0.205–1.435	0.218
	Ampullary/duodenal versus other	1.739	0.599–5.046	0.309
	dCCA versus other	0.537	0.055–5.269	0.593
Pancreatic texture	Hard versus soft	0.677	0.310–1.478	0.328
Use of piperacillin‐tazobactam	Yes versus no	0.469	0.200–1.100	0.082
Use of antifungal prophylaxis/therapy	Yes versus no	0.389	0.167–0.906	*0.029*
No preoperative biliary drainage				
Age	Continuous variable	0.990	0.943–1.039	0.687
Type of tumor	PDAC versus other	0.375	0.105–1.340	1.131
	Ampullary/duodenal versus other	1.171	0.279–4.921	0.830
	dCCA versus other	‐	‐	1
Pancreatic texture	Hard versus soft	0.268	0.095–0.922	*0.049*
Use of antifungal prophylaxis/therapy	Yes versus no	0.294	0.080–1.076	0.064
Preoperative biliary drainage				
BMI	Continuous variable	1.119	1.003–1.249	*0.044*
Duration of surgery	Continuous variable	1.005	1–1.009	*0.035*
Use of piperacillin‐tazobactam	Yes versus no	0.211	0.068–0.654	*0.007*
Use of antifungal prophylaxis/therapy	Yes versus no	0.424	0.120–1.501	0.183

*Note*: Italic values indicate the significative correlation.

Abbreviations: BMI, body mass index; CI, confidence interval; dCCA, distal cholangiocarcinoma; HR, hazard ratio; PDAC, pancreatic ductal adenocarcinoma.

### 3.4 Bacterial and fungal biliary contamination and correlation with postoperative outcomes

In the whole cohort, IBS for bacterial culture was always available, while mycological analysis was performed in 175 patients. As regards bacterial contamination, IBS was positive in 108 cases (52.7%), mostly with a polymicrobial culture (90/108, 83.3%). Although gram‐negative bacteria were more common (found in 90 cases out of 108, 83.3%), the most frequent isolated species was *Enterococcus faecalis* (*n* = 46, 42.6%), followed by *E. coli* (*n* = 42, 38.9%) and *Enterococcus faecium* (*n* = 27, 25%). Anaerobic germs were rarely found in IBS (*n* = 7, 6.5%).

**FIGURE 1:**
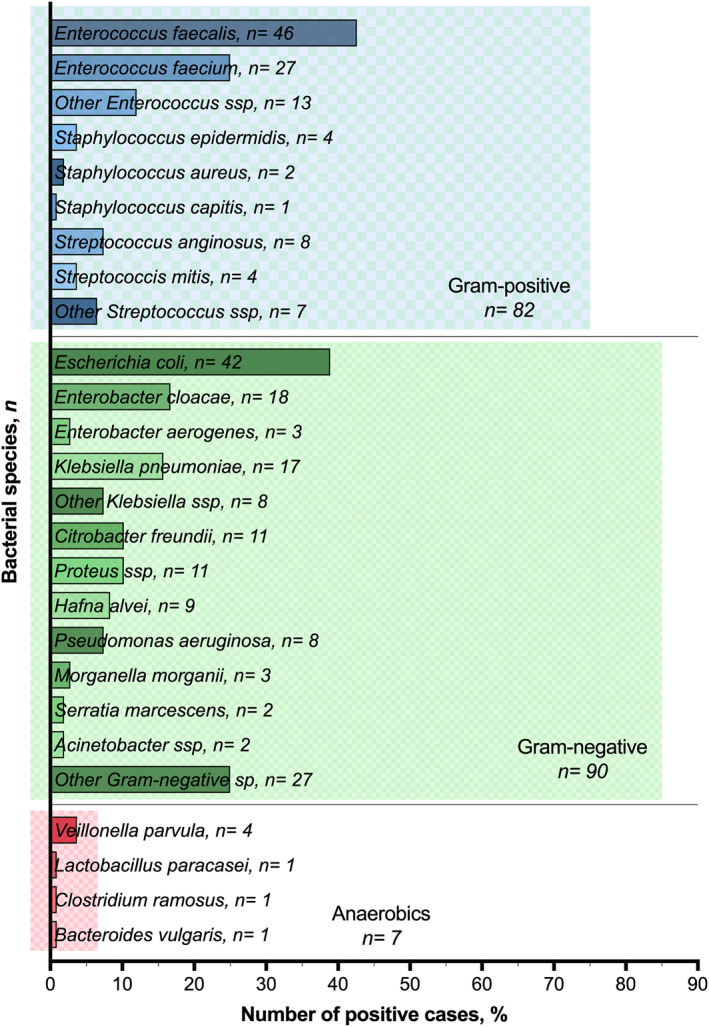
Frequencies of the different bacteria found in the intraoperative biliary samples. The *x*‐axis represents the percentage in relation to the positive cases (*n* = 108). The different germs are shown on the *y*‐axis. In each line, the name of the germ and number of cases are reported. Colors are referred to bacterial classification, with the length of the colored square in relation to the number of positive cases.

A complete summary of all bacterial species found in biliary cultures is depicted in Figure 1. Patients with a PBD had a higher risk of a polymicrobial culture (*p* < 0.001) as well as of contamination of all types of bacteria (Supporting Information S2: Online Resource 2). Species resistant to cephalosporines were more common in the PBD group compared to nondrained patients. Resistance to piperacillin‐tazobactam was less frequently found, and above all in drained patients. Comparison of main perioperative variables according to IBS positivity is shown in Supporting Information S3: Online Resource 3. Positive bacterial IBS was similar regardless of preoperative chemotherapy administration (*p* = 0.562), whereas a strong correlation with PBD and type of tumor was found (*p* < 0.001).

Positive fungal culture was found in 66 out of the 175 IBS available (37.7%). *Candida albicans* was the most common species described, being present in 75.8% of the cases with a positive fungal culture (Table 5). Resistance to fluconazole and caspofungin in infected patients was respectively found in 24% and 10% of the positive cases, respectively. Fungal biliary contamination was similarly associated with PBD (*p* < 0.001) and type of tumor (*p* = 0.005), as well as to preoperative ICU hospitalization (*p* = 0.005) (Supporting Information S3: Online Resource 3). After the analysis of postoperative outcomes, no correlation was found between bacterial and fungal contamination and the most important complications as CR‐POPF, ICs, deep or superficial SSI, or rate of major comorbidities (Supporting Information S4: Online Resource 4).

**TABLE 5:** List and frequency of fungi species found in perioperative bile samples in our cohort.

Fungi species	*n* (%)	Resistance to fluconazole, *n* (%)	Resistance to caspofungin, *n* (%)
Overall contamination	66 (100)	15 (22.7)	6 (9.1)
*Candida albicans*	50 (75.8)	2 (4)	1 (2)
*Candida glabrata*	14 (21.2)	8	1 (7.1)
*Candida tropicalis*	7 (10.6)	0	0
*Geotrichum candidum*	6 (9.1)	2 (33.3)	3 (50)
*Candida krusei*	4 (6.1)	4 (100)	0
*Candida kefyr*	4 (6.1)	1 (25)	0
*Saccharomhyces cerevisiae*	3 (4.5)	0	0
*Trichosporon*	1 (1.5)	0	1 (100)
*Other fungi*	13 (19.7)	3 (23.1)	0

*Note*: Data are shown in relation to 66 positive fungal culture out of 175 perioperative bile samples.

## 4 DISCUSSION

Despite continuous improvements in the management of pancreatic resection, perioperative antibiotic management has always been characterized by the absence of a true standardization, with significant differences in prophylaxis and therapy between different centers. Recently, a RCT demonstrated that the use of piperacillin‐tazobactam as perioperative prophylaxis reduced the postoperative SSI and POPF rate after open PD, suggesting the implementation of this therapeutic approach during pancreatic head resection.^13^ The explication lies mainly on the risk of bacterial biliary contamination which is strictly correlated with infectious and pancreatic outcomes, as largely demonstrated in literature by different series^7^, ^9^, ^10^, ^23^, ^42^ as well as by the IBS analysis of the patients included in the RCT.^14^ However, when performing subgroup analysis, the author finds out that these improvements were not evident in patients without a biliary stent, concluding that results should be interpreted with caution and may identify subgroups that benefit more or less from broad‐spectrum prophylaxis. PBD is in fact the most important independent factor associated with biliary contamination,^9^, ^18^ being a biliary stent present in more than 90% of the patients with positive bacterial IBS in our series. In this retrospective study, we confirmed that a lower rate of POPF and ICs were evident only in the presence of a PBD, while no differences were seen in the absence of a biliary stent when adding a broad‐spectrum antibiotic. Furthermore, this type of treatment was an independent predictor of POPF only in case of a biliary stent. Our study suggests therefore that piperacillin‐tazobactam before PD should be used only in case of PBD. It is worth to note, however, that in out cohort, the group piperacillin‐tazobactam includes only patients receiving at least 48 h of this antibiotic, subsequentially discontinued or modified based on the antibiogram. This is in fact part of a protocol established in our hospital, which implements the guidelines of the French Society of Anesthesiology and Intensive Care.^35^ Postoperative antibiotics are also recommended by some authors and in case of PBD by the ERAS guidelines on pancreatic surgery;^24^, ^26^, ^27^, ^43^ therefore, the comparison in this study was not between two groups with an exclusive prophylaxis. Future studies should focus on the comparison between simple prophylaxis and therapy (as in our internal protocol) in drained patients.

Another interesting data is that the 37.7% of our cohort and the 55% of the cases with a PBD had a fungal biliary contamination. The significance of this result is less explored in literature, but when assessed, a negative association with some postoperative outcomes is generally found as SSIs, overall ICs, or postoperative hemorrhage.^28^, ^31^ However, antimycotic prophylaxis or therapy during pancreatic head resection is rarely described in large series as well as outcomes according to the administration of these drugs, and no recommendation exists in antifungal care during PD. In our cohort, fungal contamination was not associated with negative postoperative outcomes. The lack of a negative effect may be the implementation of an antifungal treatment in drained patients in our protocol. This therapy reduces in fact the risk of some specific ICs in our cohort, but only in case of a previous PBD.

Finally, thanks to the implementation of a systematic IBS in our patients undergoing pancreatic head resection, we could analyze the type of germs found in biliary cultures. Our results corroborate the recent demonstration that most of the germs are resistant to cephalosporines.^14^ Furthermore, especially for gram‐negative bacteria, PBD influences not only the absolute risk of bacterial contamination but, as expected, a higher rate of antibiotic resistance, both for cephalosporine and for piperacillin‐tazobactam. To note that almost one patient out of five with a positive fungal IBS had a germ resistant to fluconazole, while being resistant to caspofungin, is rarely encountered.

Some limitations have to be reported. Firstly, although data comes from a prospective maintained database, the retrospective nature of the study limits the strength of our conclusions. Secondly, although two separate and distinct groups have been compared in terms of the antibiotic strategy, one included patients receiving a broad‐spectrum antibiotic treatment, whereas the other were cases with a narrow‐spectrum prophylaxis or treatment. Although results are clearly interpretable in light of the statistical significance, a narrow‐spectrum group made up of patients receiving exclusively prophylaxis or therapy would reinforce the value of these conclusions. Furthermore, given the multiple analysis in the subgroup of patients, some cohorts (i.e., no piperacillin/tazobactam among those without PBD) could be small and limit the results of the study. Another consideration is related to the type of pancreatic anastomosis performed. Although all patients received a pancreatico‐gastric anastomosis in our cohort, it is unknown if these results could be influenced by this type of technique and, at the same time, in case of pancreatico‐jejunostomy outcomes would have been the same. Finally, at least 48 h of antibiotics were administered in case of a prolonged treatment, but decisions based on biliary cultures results were not always standardized. As discussed in the method section in fact, after our protocol implementation in 2019, the perioperative treatment was modified or discontinued according to the presence of the antibiogram of the germs, whereas before this, the attitude was not always respected.

## AUTHOR CONTRIBUTIONS

**Fabio Giannone**: Conceptualization; data curation; formal analysis; investigation; methodology; visualization; writing–original draft. **Charles Lagarrigue**: Conceptualization; data curation; formal analysis; methodology; visualization; writing–original draft. **Oronzo Ligurgo**: Data curation; formal analysis; visualization; writing–original draft. **Lina Jazaerli**: Data curation; methodology; validation; writing–review & editing. **Paul Michel Mertes**: Conceptualization; data curation; methodology; validation; visualization; writing–review & editing. **Olivier Collange**: Conceptualization; data curation; methodology; supervision; validation; visualization; writing–review & editing. **Patrick Pessaux**: Conceptualization; data curation; formal analysis; methodology; supervision; validation; visualization; writing–review & editing

## CONFLICT OF INTEREST STATEMENT

The authors have no related conflicts of interest to declare.

## ETHICS STATEMENT

An informed consent was obtained before each procedure and the study was aligned to the ethical standards of the Helsinki declaration.

## Supporting information

Supporting Information S1

Supporting Information S2

Supporting Information S3

Supporting Information S4
