# Supplementary material for: Adaptation of antibiotics and antifungal strategy to preoperative biliary drainage to improve postoperative outcomes after pancreatic head resection
Source: World J Surg. 2024 Dec 16;49(1):270–82. doi: 10.1002/wjs.12446 (PMC11711118; doi:10.1002/wjs.12446)
Supplement: Supplementary file 1 — Supporting Information S1 [file WJS-49-270-s003.docx]

**ADAPTATION OF ANTIBIOTICS AND ANTIFUNGAL STRATEGY TO PREOPERATIVE BILIARY DRAINAGE TO IMPROVE POSTOPERATIVE OUTCOMES AFTER PANCREATIC HEAD RESECTION**

Fabio Giannone MD, PhD,^1,2,3*^ Charles Lagarrigue MD,^4*^ Oronzo Ligurgo MD,^1^ Lina Jazaerli MD,^4^ Paul Michel Mertes MD, PhD,^4^ Oliver Collange MD, PhD,^4^ Patrick Pessaux MD, PhD^1,2^

^1^ Department of Visceral and Digestive Surgery, University Hospital of Strasbourg, Strasbourg, France

^2^ Strasbourg University, Inserm, Institut de Recherche sur les Maladies Virales et Hépatiques, U1110, Strasbourg, France

^3^ Hepato-Pancreato-Biliary, Oncologic and Robotic Unit, Azienda Ospedaliero-Universitaria SS. Antonio e Biagio e Cesare Arrigo, Alessandria, Italy

^4^ Department of Anesthesiology and Intensive Care, University Hospital of Strasbourg, Strasbourg, France.

^*^ These authors share the first authorship

**Corresponding Author:**

Fabio Giannone, MD, PhD

Department of Visceral and Digestive Surgery, University Hospital of Strasbourg

1, Place de l'hôpital

Nouvel Hôpital Civil

67100 Strasbourg, France

Phone number: +33 (0) 369550552

Email: giannone.cf@gmail.com

**Online Resource 1.** STROBE flow-chart of the inclusion process

**Patients assessed for eligibility**

PD or TP for periampullary tumor between 2014 and 2022

(n= 252)

**Patients excluded**

(n= 47)

- Metastatic patients (n= 4)
- Died within 48h for non-infectious

complications (n= 6)

- IBS not performed (n= 25)
- No information on perioperative antibiotic administered (n= 12)

**Patients included in the study**

(n= 205)

- 196 PD (95.6%)
- 9 TP (4.4%)

Need for a BD before surgery n= 127 (62%)

Upfront surgery n= 78 (38%)

PD: Pancreaticoduodenectomy

TP: Total Pancreatectomy

IBS: Intraoperative bile sample

BD: Biliary drainage
