# Supplementary material for: Adaptation of antibiotics and antifungal strategy to preoperative biliary drainage to improve postoperative outcomes after pancreatic head resection
Source: World J Surg. 2024 Dec 16;49(1):270–82. doi: 10.1002/wjs.12446 (PMC11711118; doi:10.1002/wjs.12446)
Supplement: Supplementary file 2 — Supporting Information S2 [file WJS-49-270-s004.docx]

**ADAPTATION OF ANTIBIOTICS AND ANTIFUNGAL STRATEGY TO PREOPERATIVE BILIARY DRAINAGE TO IMPROVE POSTOPERATIVE OUTCOMES AFTER PANCREATIC HEAD RESECTION**

Fabio Giannone MD, PhD,^1,2,3*^ Charles Lagarrigue MD,^4*^ Oronzo Ligurgo MD,^1^ Lina Jazaerli MD,^4^ Paul Michel Mertes MD, PhD,^4^ Oliver Collange MD, PhD,^4^ Patrick Pessaux MD, PhD^1,2^

^1^ Department of Visceral and Digestive Surgery, University Hospital of Strasbourg, Strasbourg, France

^2^ Strasbourg University, Inserm, Institut de Recherche sur les Maladies Virales et Hépatiques, U1110, Strasbourg, France

^3^ Hepato-Pancreato-Biliary, Oncologic and Robotic Unit, Azienda Ospedaliero-Universitaria SS. Antonio e Biagio e Cesare Arrigo, Alessandria, Italy

^4^ Department of Anesthesiology and Intensive Care, University Hospital of Strasbourg, Strasbourg, France.

^*^ These authors share the first authorship

**Corresponding Author:**

Fabio Giannone, MD, PhD

Department of Visceral and Digestive Surgery, University Hospital of Strasbourg

1, Place de l'hôpital

Nouvel Hôpital Civil

67100 Strasbourg, France

Phone number: +33 (0) 369550552

Email: giannone.cf@gmail.com

**Online Resource** **2**. Differences on the type of positive contamination. Number of positive cases, cases resistant to cephalosporins and to piperacillin-tazobactam are compared in relation to the presence of a preoperative biliary drainage.

| **Bacterial species** | **Number of positive cases,**  ***n (%)*** | | ***p*** | **Resistance to Cephalosporins,**  ***n (%)*** | | **Resistance to Piperacillin-Tazobactam,**  ***n (%)*** | |
| --- | --- | --- | --- | --- | --- | --- | --- |
|  | *No PBD,*  *n= 78* | *PBD*  *n= 127* |  | *No PBD,*  *n= 78* | *PBD*  *n= 127* | *No PBD,*  *n= 78* | *PBD*  *n= 127* |
| Polymicrobial culture | 7 (9) | 83 (65.4) | ***<0.001*** |  |  |  |  |
| Gram-Positive Cocci | 9 (11.5) | 73 (57.5) | ***<0.001*** | 5 (6.4) | 51 (40) | 2 (2.6) | 13 (10.2) |
| Gram-Negative Bacteria | 6 (7.7) | 84 (66.1) | ***<0.001*** | - | 27 (21.2) | - | 15 (11.8) |
| Anaerobic Bacteria | - | 7 (5.5) | ***0.033*** | - | 1 (0.8) | - | 1 (0.8) |
