# Supplementary material for: Adaptation of antibiotics and antifungal strategy to preoperative biliary drainage to improve postoperative outcomes after pancreatic head resection
Source: World J Surg. 2024 Dec 16;49(1):270–82. doi: 10.1002/wjs.12446 (PMC11711118; doi:10.1002/wjs.12446)
Supplement: Supplementary file 3 — Supporting Information S3 [file WJS-49-270-s002.docx]

**ADAPTATION OF ANTIBIOTICS AND ANTIFUNGAL STRATEGY TO PREOPERATIVE BILIARY DRAINAGE TO IMPROVE POSTOPERATIVE OUTCOMES AFTER PANCREATIC HEAD RESECTION**

Fabio Giannone MD, PhD,^1,2,3*^ Charles Lagarrigue MD,^4*^ Oronzo Ligurgo MD,^1^ Lina Jazaerli MD,^4^ Paul Michel Mertes MD, PhD,^4^ Oliver Collange MD, PhD,^4^ Patrick Pessaux MD, PhD^1,2^

^1^ Department of Visceral and Digestive Surgery, University Hospital of Strasbourg, Strasbourg, France

^2^ Strasbourg University, Inserm, Institut de Recherche sur les Maladies Virales et Hépatiques, U1110, Strasbourg, France

^3^ Hepato-Pancreato-Biliary, Oncologic and Robotic Unit, Azienda Ospedaliero-Universitaria SS. Antonio e Biagio e Cesare Arrigo, Alessandria, Italy

^4^ Department of Anesthesiology and Intensive Care, University Hospital of Strasbourg, Strasbourg, France.

^*^ These authors share the first authorship

**Corresponding Author:**

Fabio Giannone, MD, PhD

Department of Visceral and Digestive Surgery, University Hospital of Strasbourg

1, Place de l'hôpital

Nouvel Hôpital Civil

67100 Strasbourg, France

Phone number: +33 (0) 369550552

Email: giannone.cf@gmail.com

**Online Resource 3.** Preoperative variables comparison between patients with and without bacterial (*n*= 205)/fungal (*n*= 175) biliary contamination

| **Variable** | No bacterial contamination, *n*= 97 | Bacterial contamination,  *n*= 108 | *p* | No fungal contamination, n= 109 | Fungal contamination, n= 66 | *p* |
| --- | --- | --- | --- | --- | --- | --- |
|  | *n (%)* | |  | *n (%)* | |  |
| Gender  Male  Female | 63 (64.9)  34 (35.1) | 64 (59.3)  44 (40.7) | 0.402 | 70 (64.2)  39 (35.8) | 39 (59.1)  27 (40.9) | 0.605 |
| BMI  <25  ≥25 | 48 (49.5)  49 (50.5) | 61 (56.5)  47 (43.5) | 0.316 | 58 (53.2)  51 (46.8) | 34 (51.5)  32 (48.5) | 0.951 |
| Weight loss >10%  No  Yes | 68 (70.1)  29 (29.9) | 83 (76.9)  25 (23.1) | 0.273 | 82 (75.2)  27 (24.8) | 51 (77.3)  15 (22.7) | 0.901 |
| ASA  I  II  III | 11 (11.3)  49 (50.5)  37 (38.1) | 3 (2.8)  54 (50)  51 (47.2) | ***0.039*** | 7 (6.4)  52 (47.7)  50 (45.9) | 2 (3)  35 (53)  29 (43.9) | 0.552 |
| Diabetes  No  Yes | 71 (73.2)  26 (26.8) | 67 (62)  41 (38) | 0.089 | 77 (70.6)  32 (29.4) | 40 (60.6)  26 (39.4) | 0.230 |
| Respiratory diseases  No  Yes | 82 (84.5)  15 (15.5) | 92 (85.2)  16 (14.8) | 0.897 | 91 (83.5)  18 (16.5) | 54 (81.8)  12 (18.2) | 0.939 |
| Cardiovascular diseases  No  Yes | 50 (51.5)  47 (48.5) | 43 (39.8)  65 (60.2) | 0.092 | 49 (45)  60 (55) | 31 (47)  35 (53) | 0.918 |
| Chronic renal failure  No  Yes | 88 (90.7)  9 (9.3) | 95 (88)  13 (12) | 0.524 | 96 (88.1)  13 (11.9) | 60 (90.9)  6 (9.1) | 0.739 |
| Preoperative ICU hospitalization  No  Yes | 93 (95.9)  4 (4.1) | 100 (92.6)  8 (7.4) | 0.317 | 108 (99.1)  1 (0.9) | 59 (89.4)  7 (10.6) | ***0.005*** |
| Preoperative antibiotics  No  Yes | 89 (91.8)  8 (8.2) | 77 (71.3)  31 (28.7) | ***<0.001*** | 96 (88.1)  13 (11.9) | 42 (63.6)  24 (36.4) | ***<0.001*** |
| Neoadjuvant therapy  No  Yes | 82 (84.5)  15 (15.5) | 88 (81.5)  20 (18.5) | 0.562 | 90 (82.6)  19 (17.4) | 54 (81.8)  12 (18.2) | 1 |
| Preoperative biliary drainage  No  Yes | 68 (70.1)  29 (29.9) | 9 (8.3)  99 (91.7) | ***<0.001*** | 57 (52.3)  52 (47.7) | 2 (3)  64 (97) | ***<0.001*** |
| Type of resection  PD  TP | 91 (93.8)  6 (6.2) | 105 (97.2)  3 (2.8) | 0.234 | 104 (95.4)  5 (4.6) | 63 (95.5)  3 (4.5) | 1 |
| Type of tumor  PDAC  Ampullary/Duodenal  dCCA  Other | 53 (54.6)  15 (15.5)  2 (2.1)  27 (27.8) | 67 (62)  29 (26.9)  8 (7.4)  4 (3.7) | ***<0.001*** | 61 (56)  20 (18.3)  6 (5.5)  22 (20.2) | 44 (66.7)  17 (25.8)  4 (6.1)  1 (1.5) | ***0.005*** |
| Pancreatic texture*  Soft  Hard | 49 (55.1)  40 (44.9) | 55 (53.4)  48 (46.6) | 0.932 | 56 (53.8)  48 (46.2) | 32 (52.5)  29 (47.5) | 0.991 |
| DOS (min), median (IQR) | 540 (470-632) | 560 (480-600) | 0.731 | 540 (430-600) | 540 (480-600) | 0.365 |
| EBL (mL), median (IQR) | 450 (250-800) | 400 (290-625) | 0.595 | 440 (300-700) | 400 (212-585) | 0.667 |

BMI: Body Mass Index; ASA: American Society of Anesthesiologists; ICU: Intensive Care Unit; PD: Pancreatoduodenectomy; TP: Total Pancreatectomy; PDAC: Pancreatic Ductal Adenocarcinoma; dCCA: distal Cholangiocarcinoma; DOS: Duration of Surgery; EBL: Estimated blood loss.

* After excluding total pancreatectomies, 4 and 2 cases missing when comparing bacterial and fungal contamination, respectively.
