# Supplementary material for: Adaptation of antibiotics and antifungal strategy to preoperative biliary drainage to improve postoperative outcomes after pancreatic head resection
Source: World J Surg. 2024 Dec 16;49(1):270–82. doi: 10.1002/wjs.12446 (PMC11711118; doi:10.1002/wjs.12446)
Supplement: Supplementary file 4 — Supporting Information S4 [file WJS-49-270-s001.docx]

**ADAPTATION OF ANTIBIOTICS AND ANTIFUNGAL STRATEGY TO PREOPERATIVE BILIARY DRAINAGE TO IMPROVE POSTOPERATIVE OUTCOMES AFTER PANCREATIC HEAD RESECTION**

Fabio Giannone MD, PhD,^1,2,3*^ Charles Lagarrigue MD,^4*^ Oronzo Ligurgo MD,^1^ Lina Jazaerli MD,^4^ Paul Michel Mertes MD, PhD,^4^ Oliver Collange MD, PhD,^4^ Patrick Pessaux MD, PhD^1,2^

^1^ Department of Visceral and Digestive Surgery, University Hospital of Strasbourg, Strasbourg, France

^2^ Strasbourg University, Inserm, Institut de Recherche sur les Maladies Virales et Hépatiques, U1110, Strasbourg, France

^3^ Hepato-Pancreato-Biliary, Oncologic and Robotic Unit, Azienda Ospedaliero-Universitaria SS. Antonio e Biagio e Cesare Arrigo, Alessandria, Italy

^4^ Department of Anesthesiology and Intensive Care, University Hospital of Strasbourg, Strasbourg, France.

^*^ These authors share the first authorship

**Corresponding Author:**

Fabio Giannone, MD, PhD

Department of Visceral and Digestive Surgery, University Hospital of Strasbourg

1, Place de l'hôpital

Nouvel Hôpital Civil

67100 Strasbourg, France

Phone number: +33 (0) 369550552

Email: giannone.cf@gmail.com

**Online Resource 4.** Post-operative variables comparison between patients with and without bacterial (*n*= 205) and fungal (*n*= 175) biliary contamination

| **Variable** | No bacterial contamination, *n*= 97 | Bacterial contamination,  *n*= 108 | p | No fungal contamination, n= 109 | Fungal contamination, n= 66 | p |
| --- | --- | --- | --- | --- | --- | --- |
|  | *n (%)* | |  | *n (%)* | |  |
| Major complications (CD>2)  No  Yes | 72 (74.2)  25 (25.8) | 80 (74.1)  28 (25.9) | 0.980 | 81 (74.3)  28 (25.7) | 52 (78.8)  14 (21.2) | 0.625 |
| Post-operative death  No  Yes | 93 (93.9)  4 (4.1) | 102 (94.4)  6 (5.6) | 0.635 | 106 (97.2)  3 (2.8) | 62 (93.9)  4 (6.1) | 0.494 |
| Overall infectious complications  No  Yes | 48 (49.5)  49 (50.5) | 64 (59.3)  44 (40.7) | 0.160 | 59 (54.1)  50 (45.9) | 38 (57.6)  28 (42.4) | 0.774 |
| Overall SSI  No  Yes | 73 (75.3)  24 (24.7) | 87 (80.6)  21 (19.4) | 0.360 | 81 (74.3)  28 (25.7) | 57 (86.4)  9 (13.6) | 0.089 |
| Type of SSI  Superficial SSI  Deep SSI | 15 (15.5)  15 (15.5) | 16 (14.8)  13 (12) | 0.897  0.476 | 16 (14.7)  17 (15.6) | 8 (12.1)  5 (7.6) | 0.803  0.188 |
| CR-POPF*  No  Yes | 67 (73.6)  24 (26.4) | 84 (80)  20 (20) | 0.375 | 79 (76)  25 (24) | 52 (82.5)  11 (17.5) | 0.419 |
| POH  No  Yes | 78 (80.4)  19 (19.6) | 90 (83.3)  18 (16.7) | 0.587 | 88 (80.7)  21 (19.3) | 58 (87.9)  8 (12.1) | 0.307 |
| Bacteriemia  No  Yes | 77 (79.4)  20 (20.6) | 90 (83.3)  18 (16.7) | 0.467 | 90 (82.6)  19 (17.4) | 55 (83.3)  11 (16.7) | 1 |
| CVC infection  No  Yes | 89 (91.8)  8 (8.2) | 96 (88.9)  12 (11.1) | 0.650 | 101 (92.7)  8 (7.3) | 61 (92.4)  5 (7.6) | 1 |
| Postoperative septic shock  No  Yes | 84 (86.6)  13 (13.4) | 95 (88)  13 (12) | 0.934 | 97 (89)  12 (11) | 56 (84.8)  10 (15.2) | 0.571 |

CD: Clavien-Dindo; SSI: Surgical site infection; CR-POPF: clinically-relevant post-operative pancreatic fistula; POH: post-operative hemorrhage, CVC: central venous catheter.

* Calculating excluding total pancreatectomies
